# Inferior ST-Segment Elevation Can Predict In-Hospital Mortality in Patients with Anterior Myocardial Infarction Complicated by Ventricular Septal Rupture

**DOI:** 10.1155/2022/7067420

**Published:** 2022-07-15

**Authors:** Xiaojuan Fan, Shun Wang, Ping Liu, Ling Bai

**Affiliations:** Department of Cardiovascular Medicine, The First Affiliated Hospital of Xi'an Jiaotong University, Shaanxi, China

## Abstract

**Background:**

Electrocardiogram (ECG) findings in patients with anterior wall myocardial infarction (MI) complicated by ventricular septal rupture (VSR) have rarely been studied.

**Methods:**

We conducted a single-center retrospective study among patients with anterior MI complicated by VSR over the past ten years. The presence of ST-segment elevation (STE) in inferior leads and new onset of complete right bundle branch block (RBBB) on ECG were studied for the prediction of in-hospital mortality.

**Results:**

Among the 85 patients enrolled, 45 (52.9%) were male, with a median age of 70 years. Sixty-five patients (76.5%) died in the hospital, and the remaining 20 patients (23.5%) had improved conditions and were discharged. Inferior STE was present in 35 patients (41.2%), including 32 patients in the death group and 3 patients in the survival group (*P* = 0.005). New-onset RBBB was present in 25 patients (29.4%), with 22 patients in the death group and 3 patients in the survival group (*P* = 0.103). Multivariate logistic regression showed that inferior STE was an independent predictor of in-hospital death in patients with anterior MI and VSR (OR: 14.488; 95% CI: 1.708-122.887; *P* = 0.014).

**Conclusions:**

In patients with anterior MI complicated by VSR, inferior STE was associated with a higher risk of in-hospital mortality.

## 1. Introduction

Ventricular septal rupture (VSR) is a catastrophic complication after acute myocardial infarction (AMI). Patients with postinfarction VSR have a high mortality rate even in the contemporary era. The ESC guideline suggests delayed repair if patients can be effectively stabilized by aggressive therapy [1]. Considered delayed surgery allows easier septal repair in scarred tissue when the myocardial edema subsides, but it would increase the risk of rupture extension and lead to death while the patients are waiting for surgery. The AHA guideline recommends early surgery even in hemodynamically stable patients [2]. However, two recent studies revealed that early surgery was associated with a low survival rate, and operative mortality has remained unchanged over the last 2 decades [3, 4]. Other factors associated with worse outcomes include older age, female sex, anterior AMI, no history of angina or myocardial infarction, and Killip class III or IV [5–7]. However, the above predictors are all based on the clinical profile; few studies have explored the prognostic value of electrocardiogram (ECG) characteristics in patients with postinfarction VSR.

VSR may occur within 24 h to several days after MI and with equal frequency in patients with anterior and posterolateral MI. The rupture site in anterior MI is mostly located at the ventricular septum near the apex [8]. The blood supply for the ventricular apex can be double supplied by the left anterior descending artery (LAD) and the right coronary artery (RCA) or provided by the LAD alone, which is called wrap-around LAD. A wrap-around LAD is defined as an LAD reaching beyond the apex to supply the apex and part of the inferior wall. Acute occlusion of a wrap-around LAD or acute occlusion of a non-wrap-around LAD following prior occlusion of the RCA may result in ST-segment elevation (STE) not only in anterior leads but also in inferior leads [9, 10]. In this situation, the blood supply to the apical myocardium is totally interrupted, resulting in transmural necrosis and a higher risk of perforation.

Another ECG feature associated with increased mortality in patients with anterior MI is the new onset of complete right bundle branch block (RBBB). The presence of RBBB in the setting of anterior MI indicates a large infarct area and serves as a significant independent predictor of high in-hospital mortality [11, 12].

Therefore, the purpose of our study was to investigate the predictive value of inferior STE and new-onset RBBB for in-hospital mortality in patients with anterior MI combined with VSR.

## 2. Methods

### 2.1. Study Design and Patients

This was a single-center, retrospective study conducted among AMI patients in the cardiovascular department of the First Affiliated Hospital of Xi'an Jiaotong University from January 2011 to April 2021. Patients with anterior AMI complicated by VSR were screened and enrolled. A diagnosis of anterior AMI met all of the following components: clinical symptoms of chest pain, elevated cardiac troponin, pathological Q-wave on precordial lead, and abnormal anterior wall motion on 2-dimensional echocardiography. VSR was confirmed by physical examination of a loud systolic murmur and echocardiography with the presence of a blood flow signal across the ventricular septum detected by color Doppler. The study was approved by the ethics committee of our institution. Written informed consent was waived due to the retrospective design.

### 2.2. Clinical Data Collection

The following information from the patients' clinical records was collected for the purpose of our study: age; sex; previous history of hypertension and diabetes mellitus (DM); time from symptom onset to admission (classified into ≤12 h, 12 h~48 h, 3 d~7 d, and >7 d); Killip class at the time of VSR confirmation; laboratory results including serum alanine transaminase (ALT), creatinine (CRE), and N-terminal probrain natriuretic peptide (NT-proBNP) levels; any procedure including coronary angiography (CAG), primary percutaneous coronary intervention (PCI), surgical repair, or transcatheter closure for VSR; and coronary artery bypass grafting (CABG) during hospitalization.

Echocardiography was used to acquire the left ventricular ejection fraction (LVEF) measured by the Simpson method and the size and location of the VSR. The presence of left ventricular aneurysm and pericardial effusion was recorded if they existed.

ECG characteristics were identified by two experienced doctors. Inferior STE was defined as an ST-segment elevation ≥ 1.0 mm in at least two continuous inferior leads (II, III, and aVF). New-onset RBBB was defined as the new development of RBBB associated with AMI, and preexisting RBBB was not considered.

All enrolled patients were divided into two groups according to in-hospital death: the death group and the survival group.

### 2.3. Statistical Analysis

Analyses were performed by SPSS version 25.0. Continuous variables are presented as median values with interquartile ranges (IQRs), and categorical variables are presented as counts and frequencies. Differences between the two groups were compared by the use of the Mann–Whitney test for continuous variables and the *χ*^2^ test or Fisher's exact test for categorical variables as appropriate. Binary logistic regression analysis was used to evaluate predictors of in-hospital death. Odds ratios (ORs) and 95% confidence intervals (CIs) were calculated. Variables that were statistically significant at the univariate level were tested by multivariate analysis with forward stepwise regression to identify the independent predictors of in-hospital mortality. Two-tailed *P* < 0.05 was regarded as statistically significant in all calculations.

## 3. Results

### 3.1. Comparison of Clinical Profiles

During the study period, 15507 patients with a diagnosis of AMI were screened; among them, a total of 85 (0.55%) patients with anterior AMI complicated by VSR were included in the present study. Seventy-eight patients had VSR near the apex, and the other 7 patients had VSR at the midseptum. Twenty patients survived and were discharged (the survival group), while 65 patients died in the hospital (the death group). Baseline clinical characteristics are shown in Table 1. There was no significant difference in age, sex, history of hypertension and DM, or the value of NT-proBNP and CRE between the two groups. Compared with the survival group, the death group had a shorter time from symptom onset to admission, a higher proportion of Killip class IV, and a higher serum ALT value (all *P* < 0.05).

### 3.2. Comparison of Echocardiographic Characteristics

There was no statistical difference in LVEF, left ventricular aneurysm, pericardial effusion, VSR size, or location between the two groups (all *P* > 0.05) (Table 1).

### 3.3. Comparison of ECG Characteristics

**Figure 1:**
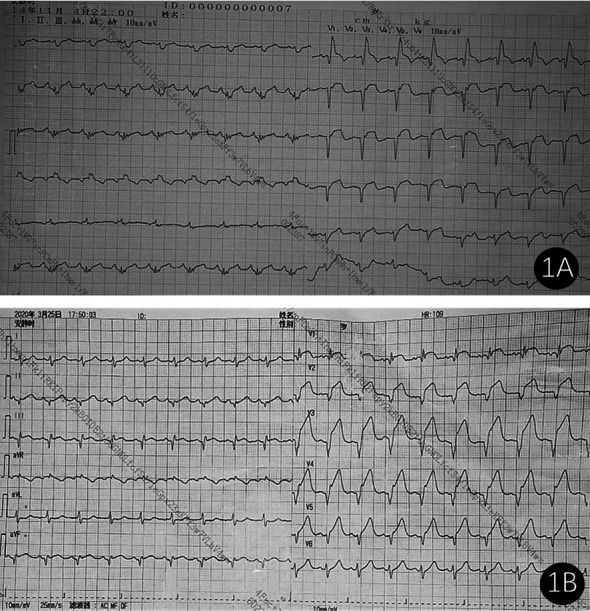
The ECG manifestations of two patients with anterior wall MI and VSR. (a) Patient 1: a 61-year-old male complained of chest pain for 3 days. ECG showed RBBB and Q-wave formation in leads V1-V4 and ST-segment elevation in leads V1-V5 and II, III, and aVF. (b) Patient 2: a female, 82 years old, complained of chest pain for 1 day. ECG showed Q-wave formation and ST-segment elevation in leads V1-V5, III, and aVF.

Of the 85 patients with anterior AMI and VSR, 35 patients presented inferior STE on ECG, including 32 patients in the death group and 3 patients in the survival group, with a significant difference (49.2% vs. 15.0%, *P* = 0.005). New-onset RBBB was noted in 25 patients, including 22 patients in the death group and 3 patients in the survival group, with no significant difference (33.8% vs. 15.0%, *P* = 0.103). The simultaneous presence of inferior STE and new-onset RBBB was noted in 8 patients, including 7 patients in the death group and 1 patient in the survival group, with no statistical difference (10.8% vs. 5.0%, *P* = 0.672) (Table 1 and Figure 1).

### 3.4. Comparison of Procedures

Among the 85 patients, 35 patients received CAG; 12 patients had single-vessel disease, 9 patients had double-vessel disease, and 14 patients had three-vessel disease. There was no difference in coronary severity between the two groups (*P* = 0.910). Primary PCI was performed for 24 patients, including 14 patients in the death group and 10 patients in the survival group (*P* = 0.017). Transcatheter closure for VSR was performed for 6 patients, and surgical repair was performed for 11 patients, with a statistical difference between the two groups (*P* < 0.001) (Table 1).

### 3.5. Predictors of In-Hospital Mortality

Univariate logistic analysis showed that inferior STE (*P* = 0.012) and Killip class IV (*P* = 0.011) were associated with a higher risk of in-hospital mortality, while a time from symptom onset to admission > 7 days (*P* < 0.05), a previous diagnosis of DM (*P* = 0.039), and primary PCI (*P* = 0.017) were associated with a lower risk of in-hospital death (Table 2).

To determine the independent predictors of in-hospital death, the above 5 variables were further analyzed by multivariate analysis using forward stepwise regression. As a result, inferior STE (*P* = 0.014) and Killip class IV (*P* = 0.006) were independently associated with a higher risk of in-hospital mortality in patients with anterior AMI and VSR, while a time from symptom onset to admission > 7 days (*P* < 0.05) and primary PCI (*P* = 0.025) were associated with a lower risk of in-hospital mortality (Table 3).

## 4. Discussion

Our study found that patients with anterior AMI and VSR presented a high prevalence of inferior STE and new-onset RBBB on ECG. In addition to traditional risk factors, electrocardiographic STE in inferior leads can independently predict in-hospital mortality in patients with anterior AMI and VSR.

There were only two studies investigating the ECG features in AMI patients complicated with VSR. Hayashi et al. [13] studied 21 patients with anterior AMI and VSR and found that patients presenting with inferior STE at admission were more prone to VSR. In their study, inferior STE was noted in 9 patients (42.9%), and the incidence was significantly higher in anterior AMI patients with VSR than that in anterior AMI patients without VSR (3.6%). They concluded that ECG findings could be a useful predictor for the occurrence of VSR in anterior AMI patients. Another study [14] found that the magnitude of STE in lead V2 and the magnitude of ST depression in lead III were independently associated with the occurrence of VSR following AMI. To date, there has been a lack of research analyzing ECG features in a larger group of patients with anterior AMI and VSR, especially when the incidence of postinfarction VSR is increasingly decreased in the reperfusion era.

In our study, the total incidence of inferior STE was 41.2%, which was comparable to that in Hayashi et al.'s report (42.9%) [13]. In addition to its high incidence, it was also verified to be an independent predictor of in-hospital mortality. Previous studies [10, 15] indicated that inferior STE in patients with anterior MI was due to the acute occlusion of a wrap-around LAD in most cases or due to occlusion of a non-wrap-around LAD on the basis of a chronic occluded RCA in a few cases, with the absence of collaterals. STE is a marker of acute coronary occlusion and can rapidly normalize if reperfusion is complete. For patients presenting ≤12 hours after symptom onset, simultaneous STE in anterior and inferior leads indicates a large infarct area and poor cardiac systolic function, manifested as an impaired LVEF, a faster HR, and a lower BP, leading to a poor outcome. However, for patients presenting late who are not receiving early reperfusion, the natural normalization of ST-elevation takes hours to days. Persistent STE in the precordial lead is usually considered a marker of left ventricular aneurysm. We studied the correlation between inferior STE and ventricular aneurysm but found no significance (*P* = 0.121). The 11^th^ edition of *Braunwald's Heart Disease* [16] states that persistent STE seen several weeks or more after MI correlates strongly with severe underlying wall motion disorders, although the cause is not necessarily a frank ventricular aneurysm. Another possible explanation for persistent STE is pericardial effusion due to myocardial inflammation or hemorrhagic exudation into the pericardium. At autopsy, pericardial effusion and a secondary rupture at the left ventricular free wall were confirmed to be present in a few patients with postinfarction VSR [17]. On echocardiography, a small amount of pericardial effusion was found in some of our patients, and this has also been reported in the literature [18]. Furthermore, it has been reported that AMI patients with apical VSR can experience sudden death due to secondary free wall rupture [19, 20]. Regardless of whether persistent STE is due to a ventricular aneurysm, a severe wall motion disorder, or pericardial effusion secondary to free wall rupture, these factors all indicate poor outcomes.

Another ECG feature was the high incidence of new-onset RBBB in patients with anterior AMI and VSR. In our study, there were 25 patients with new-onset RBBB, including 22 patients in the death group and 3 patients in the survival group. Although there was no significant difference between the two groups, the total incidence of new-onset RBBB was 29.4%, which was obviously higher than that in the overall population of anterior MI patients. Shrivastav et al. [11] studied more than one million patients with anterior MI and found that the incidence of new-onset RBBB was only 1.8%. The right bundle branch receives most of its blood supply from the septal branches of the LAD, particularly in its initial course. LAD occlusion proximal to the first septal branch results in a large infarct area and brings a higher risk of RBBB and septal rupture. Compared to patients without RBBB, patients with anterior MI and new-onset RBBB always have a poor prognosis [11, 12]. In our study, 8 patients presented with both inferior STE and RBBB, including 7 patients in the death group and 1 patient in the survival group, with no significant difference. A possible explanation for the lack of a difference may be the impact of the limited patient population on statistical power. As clinicians pay more attention to the role of ECG in AMI patients with VSR, these ECG characteristics could be further explored and verified.

Except for ECG features, a notable factor associated with mortality was the presentation time from symptom onset. Even in the reperfusion era, there is a high proportion of STEMI patients who are not receiving early reperfusion, especially patients living in rural areas. Delayed care seeking by AMI patients with VSR leads to two outcomes: dying at home or surviving the initial course. A longer time of admission for surviving patients may suggest that they were “self-selected” as more stable patients, which explained the better prognosis. Another factor that influenced the outcome was primary PCI. Theoretically, primary PCI can salvage the ischemic myocardium at the infarction border zone and shorten the surgical operation time with no need for simultaneous CABG. However, in the CAUTION study [3], preoperative PCI was associated with early mortality in patients undergoing surgical repair, which was not consistent with our conclusion. The underlying cause may be the discrepancy in the study subjects and the timing of PCI. Moreover, cardiogenic shock is an established factor associated with high mortality, and we obtained consistent results by analyzing the presence of Killip class IV. Overall, the above clinical features indicated the importance of early presentation (≤12 h) and primary PCI for AMI patients, since reperfusion therapy has been associated with a lower incidence of post-VSR [21]. And we believe that the sustainable development and construction of the Chest Pain Center and accessible medical services will help to reduce the number of deaths due to delayed presentation.

However, it should be mentioned that emergency surgery was performed on only a few patients in our study, and most patients died during the waiting time. As the cardiac team improves and the surgical technique progresses, the proportion of early surgery in our center will increase. Our ECG findings provide a supplement for the existing evaluation indicators. Whether the two ECG characteristics are associated with high surgical mortality needs to be further explored.

In summary, our study mainly focused on two ECG features in patients with anterior MI and VSR. Although the chance of survival for MI patients with VSR depends on surgical repair, the optimal timing is unclear. Delayed surgery is always associated with better outcomes in stable patients. It is important to identify which patients are at high risk and may gain more benefit from early surgery. The ECG findings may provide more information for disease evaluation in clinical practice.

## 5. Conclusions

In addition to clinical characteristics, the presence of inferior STE on ECG is an independent predictor of in-hospital mortality in patients with anterior MI complicated by VSR.

## Figures and Tables

**Table 1:** Comparison of baseline clinical profiles between the two groups.

	Death group (*n* = 65)	Survival group (*n* = 20)	*P* value
Age (years)	72 (63~78)	70 (62~75)	0.428
Male, *n* (%)	32 (49.2%)	13 (65.0%)	0.213
Hypertension, *n* (%)	34 (52.3%)	10 (50.0%)	0.894
Diabetes mellitus, *n* (%)	11 (16.9%)	8 (40.0%)	0.061
Time from symptom onset to admission, *n* (%)			<0.001
≤12 h	19 (29.3%)	4 (20.0%)	
12 h~48 h	20 (31.0%)	1 (5.0%)	
3 d~7 d	20 (31.0%)	3 (15.0%)	
>7 d	6 (8.6%)	12 (60.0%)	
Killip class IV, *n* (%)	32 (49.2%)	3 (15.0%)	0.007
NT-proBNP (pg/mL)	8280 (3711~19509)	4016 (2282~8189)	0.074
ALT (U/L)	74 (50-351)	32 (19-65)	0.001
CRE (*μ*mol/L)	101 (72-151)	83 (71-110)	0.171
Electrocardiography			
Inferior STE, *n* (%)	32 (49.2%)	3 (15.0%)	0.005
New-onset RBBB, *n* (%)	22 (33.8%)	3 (15.0%)	0.103
Coexistence of inferior STE and RBBB, *n* (%)	7 (10.8%)	1 (5.0%)	0.672
Echocardiography			
LVEF (%)	45 (39~51)	46 (40~49)	0.634
Left ventricular aneurysm, *n* (%)	30 (46.2%)	11 (55.0%)	0.489
Pericardial effusion, *n* (%)	7 (35.0%)	17 (26.2%)	0.442
Apical VSR, *n* (%)	19 (95.0%)	59 (90.8%)	1
VSR size (mm)	10.0 (7.0~12.8)	8.0 (6.0~12.8)	0.369
CAG (*n* = 35)			0.910
Single-vessel, *n* (%)	7 (36.8%)	5 (31.3%)	
Double-vessel, *n* (%)	5 (26.3%)	4 (25.0%)	
Multivessel, *n* (%)	7 (36.8%)	7 (43.8%)	
Primary PCI, *n* (%)	14 (21.5%)	10 (50.0%)	0.017
CABG, *n* (%)	2 (3.1%)	1 (5.0%)	0.558
Transcatheter closure/surgical repair for VSR, *n*	1/4	5/7	<0.001
Time from symptom onset to VSR repair (d)	36 (19-44)	30 (24-33)	0.115

NT-proBNP: N-terminal probrain natriuretic peptide; ALT: serum alanine transaminase; CRE: creatinine; STE: ST-segment elevation; RBBB: right bundle branch block; LVEF: left ventricular ejection fraction; VSR: ventricular septal rupture; CAG: coronary angiography; PCI: percutaneous coronary intervention; CABG: coronary artery bypass grafting.

**Table 2:** Predictors of in-hospital mortality by univariate logistic analysis.

Variables		OR (95% CI)	*P* value
Time from symptom onset to admission	>7 d	Reference	
	<12 h	9.350 (2.049-42.658)	0.004
	12 h-48 h	39.600 (4.074-384.952)	0.002
	3 d-7 d	13.200 (2.623-66.434)	0.002
Inferior STE		5.471 (1.443-20.743)	0.012
Previous DM		0.306 (0.100-0.942)	0.039
Primary PCI		0.275 (0.095-0.790)	0.017
Killip IV		5.495 (1.468-20.574)	0.011

STE: ST-segment elevation; DM: diabetes mellitus; PCI: percutaneous coronary intervention.

**Table 3:** Independent predictors of in-hospital mortality by multivariate analysis.

Variables		OR (95% CI)	*P* value
Time from symptom onset to admission	>7 d	Reference	
	<12 h	25.588 (3.030-216.087)	0.003
	12 h-48 h	58.222 (3.733-907.959)	0.004
	3 d-7 d	16.471 (1.869-145.173)	0.012
Inferior STE		14.488 (1.708-122.887)	0.014
Primary PCI		0.146 (0.027-0.784)	0.025
Killip IV		21.905 (2.427-197.701)	0.006

STE: ST-segment elevation; PCI: percutaneous coronary intervention.

## Data Availability

Any researcher can get the data from the first or corresponding author with reasonable request.
